# X-ray reflectivity theory for determining the density profile of a liquid under nanometre confinement

**DOI:** 10.1107/S0909049510014858

**Published:** 2010-05-19

**Authors:** Edith Perret, Kim Nygård, Dillip K. Satapathy, Tobias E. Balmer, Oliver Bunk, Manfred Heuberger, J. Friso van der Veen

**Affiliations:** a Paul Scherrer Institut, 5232 Villigen PSI, Switzerland; b Université Fribourg, 1700 Fribourg, Switzerland; c ETH Zürich, 8093 Zürich, Switzerland; d EMPA, 9014 St Gallen, Switzerland

**Keywords:** X-ray reflectivity, confined fluids

## Abstract

An X-ray reflectivity theory on the determination of the density profile of a molecular liquid under nanometre confinement is presented. The confinement geometry acts like an X-ray interferometer, which consists of two opposing atomically flat single-crystal mica membranes with an intervening thin liquid film of variable thickness. The X-rays reflected from the parallel crystal planes (of known structure) and the layered liquid in between them (of unknown structure) interfere with one another, making X-ray reflectivity highly sensitive to the liquid’s density profile along the confinement direction. An expression for the reflected intensity as a function of momentum transfer is given. The total structure factor intensity for the liquid-filled confinement device is derived as a sum of contributions from the inner and outer crystal terminations. The method presented readily distinguishes the confined liquid from the liquid adsorbed on the outer mica surfaces. It is illustrated for the molecular liquid tetrakis(trimethyl)siloxysilane, confined by two mica surfaces at a distance of 8.6 nm.

## Abstract 2

The determination of out-of-plane density profiles of a confined molecular liquid by synchrotron X-ray reflectivity is presented.

## 1. Introduction

The investigation of the behaviour of liquids under confinement (‘nanofluidics’) is a research topic of technological relevance. The confinement geometry directly influences lubrication and wetting properties as well as the diffusion and transport of the liquid’s constituents. Theoretical models and molecular dynamics simulations, mostly for hard-sphere liquids, predict pronounced confinement effects on the local structure of the liquid (Persson & Tosatti, 1994 ▶). They all indicate (Kjellander & Sarman, 1991 ▶; Gao *et al.*, 1997 ▶; Schoen & Dietrich, 1997 ▶) that the liquid’s molecules order in layers parallel to confining surfaces. However, they generally differ in their predictions regarding the shape of the liquid’s layered density profile along the confinement direction. Earlier X-ray reflectivity experiments confirmed the existence of the layering effect near a single flat surface (Huisman & van der Veen, 1998 ▶; Reichert *et al.*, 2000 ▶; Yu *et al.*, 1999 ▶, 2000 ▶) and evidence for a layering-induced thickness quantization effect was provided by a recent structural investigation of a liquid within a nanometre-sized gap (Seeck *et al.*, 2002 ▶). A considerable fraction of confined liquids research has been performed using the surface force apparatus (SFA) (Israelachvili & McGuiggan, 1990 ▶). As the surfaces approach each other to within a few molecular diameters, the SFA typically records oscillations in the normal force, with each oscillation representing the expulsion of a single molecular layer. Although the oscillations are suggestive of structural layering within the narrow gap, surface force studies, by their very nature, cannot reveal the liquid’s structure.

We present X-ray reflectivity (XRR) calculations for a liquid confined by two flat single crystals of known structure. Interferences between the amplitudes scattered from the opposing crystal lattice planes and the density variations of the liquid provide high sensitivity to the liquid’s density profile across the gap. The structure of the liquid is found by searching for the best fit between model-dependent theoretical reflectivity curves and experimental ones. We demonstrate the method for tetrakis(trimethyl)siloxysilane (TTMSS) confined by two cleaved mica membranes.

The paper is organized as follows. §2 introduces the confinement device and provides details about the X-ray reflectivity experiment. §3 presents a calculation of the total structure factor for the confinement device (crystals and liquid). The total structure factor is decomposed into partial structure factors from the confining crystals, the confined liquid and possible liquid layers adsorbed on the outer crystal surfaces. §4 presents calculations illustrating that the structure of confined and adsorbed liquid are distinguishable and compares a measured reflectivity curve with theoretical ones through a fitting procedure revealing the liquid’s density profile. A conclusion and outlook are presented in §5.

## 2. Experiment

**Figure 1:**
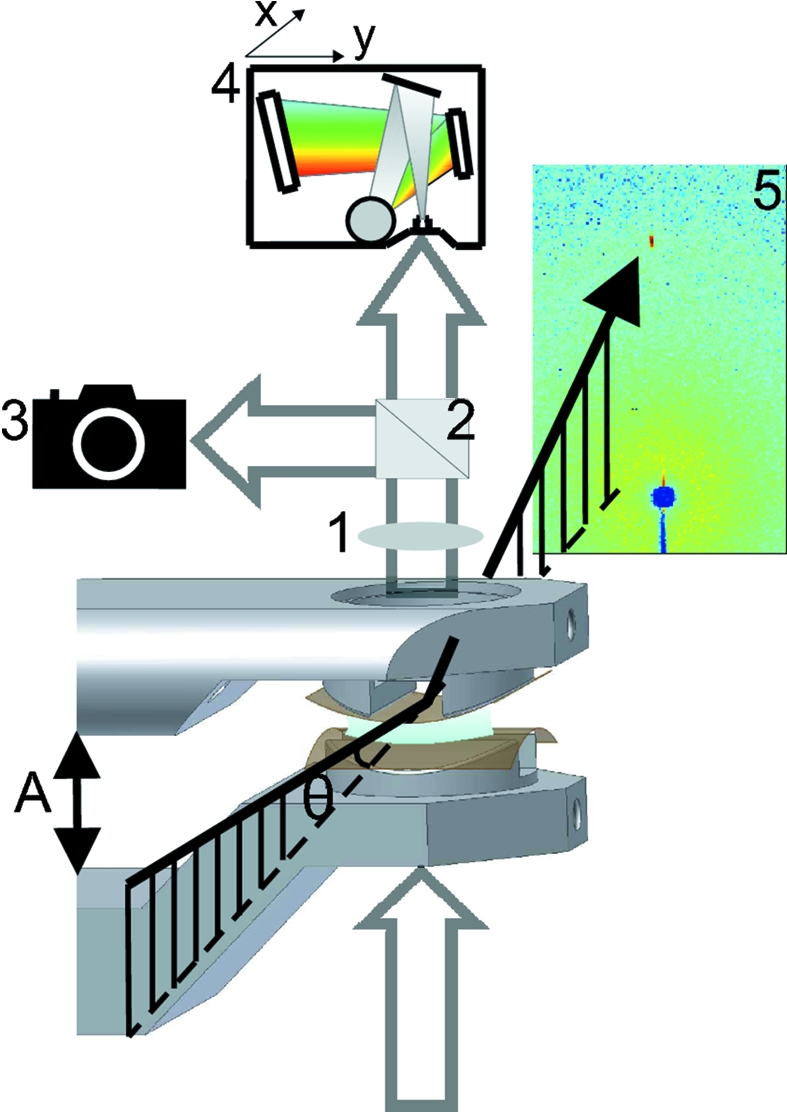
Schematic representation of a specular X-ray reflectivity experiment on a confined liquid. The gap width is controlled by the actuator *A* and measured through white-light interferometry. A focusing lens (1) is positioned after the interferometer and a beam splitter (2) directs the light to a CCD camera (3) and to a spectrometer (4). The momentum-transfer dependence of the reflected intensity is measured in a θ–2θ scan, where the scattered beam is detected by a PILATUS 100K (Kraft *et al.*, 2009 ▶) detector (5) having a pixel size of 172 µm × 172 µm.

The liquid was confined within an extended surface force apparatus (eSFA) which was modified for X-ray reflectivity experiments (Fig. 1 ▶). In contrast to the original eSFA (Heuberger, 2001 ▶; Heuberger *et al.*, 2001 ▶), the modified device does not allow for calibrated force measurements but merely serves to obtain flat confined liquid films over an area of some hundreds of micrometres in diameter. The mica membranes confining the liquid medium represent an optical interferometer (Born & Wolf, 1980 ▶) similar to the one used in the SFA but without solid support and metal mirrors. An actuator allows for accurately tailoring the distance between the confining crystals while the distance is continuously monitored by white-light interferometry (Israelachvili, 1971 ▶; Heuberger, 2001 ▶). White light is directed through the interferometer onto the pinhole aperture of the spectrometer. Each acquired transmission spectrum results from a single spot within the contact area. The local distance between the mica membranes is determined from the transmission spectrum using the fast spectral correlation method (Heuberger, 2001 ▶; Israelachvili, 1973 ▶) and multilayer matrix method (Born & Wolf, 1980 ▶; Clarkson, 1989 ▶). The spectrometer is mounted on an *xy*-stage. Through scanning of the spectrometer in the *xy*-plane, lateral variations in the thickness of the confined film can be detected immediately.

**Figure 2:**
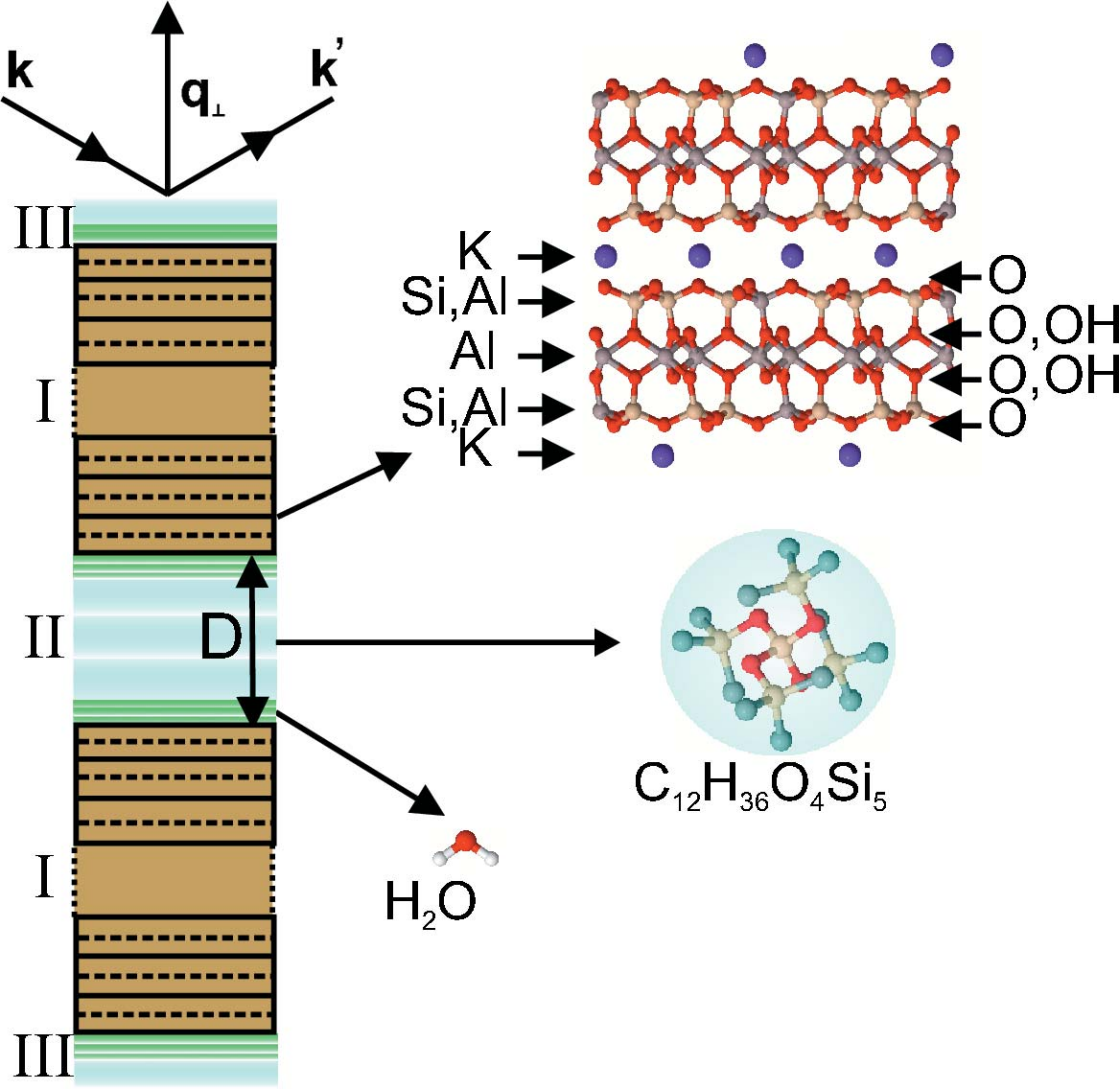
Confinement geometry. Left-hand side: stack, (I) single-crystal membranes of mica with *N*_3_ unit cells, (II) liquid in the gap and (III) condensed liquid on the outer mica surfaces. Right-hand side: molecular structures of muscovite mica, TTMSS and water. The gap width *D* is defined as the distance between the surface potassium ions of the opposing mica crystals.

A symmetric planar confinement geometry was obtained as follows. A thin single-crystal mica membrane of (001) orientation was cleaved from a large crystal. The micrometre-thin membrane was then cut into two pieces, which were glued onto Invar cylindrical supports with their freshly cleaved faces being exposed. Both supports have rectangular areas cut out, leaving the central part of the membranes unsupported. The two crystals were brought to close proximity and a liquid droplet, in this study TTMSS, was inserted using a syringe. In order to avoid fast evaporation of the liquid the vapour pressure in the chamber was increased by a TTMSS reservoir in a small cuvette. The non-zero vapour pressure gave rise to condensed TTMSS layers on the water-covered outer mica surfaces. Upon fast approach, liquid became trapped and a pocket was formed, which slowly drained out until a flat layered film of typically 300 µm × 300 µm in size was obtained (Perret *et al.*, 2009 ▶). The crossed pair of free-standing mica membranes with liquid in between was aligned such that the focused beam impinges onto the centre of the flat confined film area, which made it possible to measure the X-ray reflectivity from an oriented planar mica–liquid–mica stack. The stack can be regarded as a single crystal having an extended planar vacancy of adjustable thickness which is filled with liquid (Fig. 2 ▶). The assumption of a symmetric geometry is justified by the fact that the mica sheets have the same thickness and that they are surrounded by the same gas environment.

Muscovite mica [H_2_KAl_3_(SiO_4_)_3_] is a stack of aluminium silicate sheets separated by sheets of potassium ions in the (001) plane. After cleavage along a (001) plane, the exposed surface is assumed to be terminated with ∼1/2 monolayer of potassium ions. The crystal unit cell is monoclinic (Güven, 1971 ▶) with dimensions *a* = 5.19 Å, *b* = 9.01 Å and 2*c* = 20.05 Å with β = 95.76°. The unit cell contains *c*-glide and *n*-glide planes as symmetry elements, therefore the structure is repeated by half of the mica unit cell *c*. For the following calculations the term ‘mica unit cell’ is used for the vertically repeated volume spanned by the vectors **a**, **b** and **c**. The X-ray scattering experiment was performed at the cSAXS beamline (X12SA) of the Swiss Light Source at the Paul Scherrer Institut, Villigen. A photon wavelength of 0.75 Å (energy 16.5 keV) was selected. The specular X-ray reflectivity as a function of perpendicular momentum transfer *q*_⊥_ was measured as follows. A PILATUS 100K pixel detector (Kraft *et al.*, 2009 ▶) was positioned at a distance *R* = 0.46 m from the centre of the confined film. The total number of photons in the specular reflection (‘integrated intensity’) (Vlieg, 1997 ▶) was determined by integrating the scattered intensity *I*_s_(**q**) over the exposure time *T* and the receiving detector area *A*_det_ at the position of the reflection,
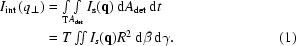
Here, **q** is the momentum transfer given by **k**′ − **k**, with **k**′ the exit wavevector and **k** the incident wavevector. The angles β and γ are angular integration variables over the detector area and *R* is the distance from the centre of the confined film to the detector. An intrinsic background arising from diffuse scattering was measured next to the specular reflectivity spots and subtracted from the integrated intensity.

From the measured specularly reflected integrated intensity we derive the modulus of the structure factor (‘structure factor amplitude’) for the entire confinement device through use of the relation (Vlieg, 1997 ▶)
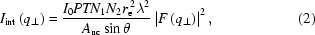
where *I*_0_ is the incident number of photons per second and unit area, *P* the polarization factor, *T* the exposure time, *N*_1_ *N*_2_ the number of illuminated surface mica unit cells, *r*_e_ the classical electron radius, λ the wavelength, *A*_uc_ the in-plane unit cell area and θ the angle of incidence of the beam. The structure factor *F*(*q*_⊥_) in (2) refers to *A*_uc_. The Lorentz factor sinθ corrects for the elongated intercept of the crystal truncation rod (Robinson, 1986 ▶) with the Ewald sphere, provided the intercepted angular range is fully captured by the analyzing window of the detector (Torrelles & Rius, 2004 ▶). The polarization *P* is equal to 1 in our specular reflection geometry. Denoting the horizontal and vertical beam size at the sample by *L*_h_ and *L*_v_, we can write *N*_1_ *N*_2_ = *f* *L*_h_ *L*_v_/(*A*_uc_sinθ), where the correction factor *f* takes into account that the beam cross section is not accurately known and that only part of the beam might fall onto the planar confined film area (ideally, *f* ≃ 1). We therefore obtain 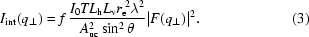
All the pre-factors of |*F*(*q*_⊥_)|^2^ are known except for the correction factor *f*, which is used as a fitting parameter with its initial value set to 1. The measured *I*_int_(*q*_⊥_) values therefore yield a set of experimental |*F*_exp_(*q*_⊥_)| values. These will be compared with structure factors |*F*_calc_(*q*_⊥_)| calculated for a variety of liquid structure models in a search for the best fit.

The confined liquid TTMSS is prone to radiation damage. Below we provide an estimate of the irradiation dose *D*_abs_ in our experiment in units of Gray (Gy), where 1 Gy is the absorption of 1 J of radiation energy by 1 kg of matter. For the irradiation dose we therefore have 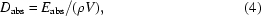
where *E*_abs_ is the absorbed photon energy, ρ is the mass density of the material and *V* is the irradiated volume. The absorbed energy is given by *E*_abs_ = *N* *h*ν{1 − exp[−(μ_en_/ρ)ρ*L*_p_]}, where *N* is the total incident number of photons of energy *h*ν, μ_en_/ρ is the tabled mass energy-absorption coefficient (Seltzer, 1993 ▶) and *L*_p_ is the path length of the beam through the material. For a thin sample, *E*_abs_ ≃ *Nh*ν(μ_en_/ρ)ρ*L*_p_. Using *V* = *L*_p_ *L*_h_ *L*_v_ we can rewrite (4) as 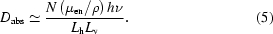
Taking weighted averages of the mass energy-absorption coefficients over the mica unit cell and the TTMSS molecule we find (μ_en_/ρ)_mica_ = 5.5 cm^2^ g^−1^ and (μ_en_/ρ)_TTMSS_ = 3.3 cm^2^ g^−1^ at a photon energy of 16.5 keV. For the reflectivity scan shown in §4 the total number of incident photons was *N* = 5.2 × 10^11^ for an irradiation time of 140 s. With *L*_h_ × *L*_v_ being 147 µm × 10 µm, we find irradiation doses of (*D*_abs_)_mica_ = 0.5 MGy and (*D*_abs_)_TTMSS_ = 0.3 MGy. The average absorbed energy in one TTMSS molecule corresponds to 1.2 eV. The applied dose during the experiment has not led to noticeable radiation damage, as we have verified by repeating part of the reflectivity scans. In addition, the confined TTMSS was found to have retained its liquid properties after the experiment, indicating that cross-linking of molecules has not taken place to a noticeable extent.

## 3. Calculation of total structure factor

**Figure 3:**
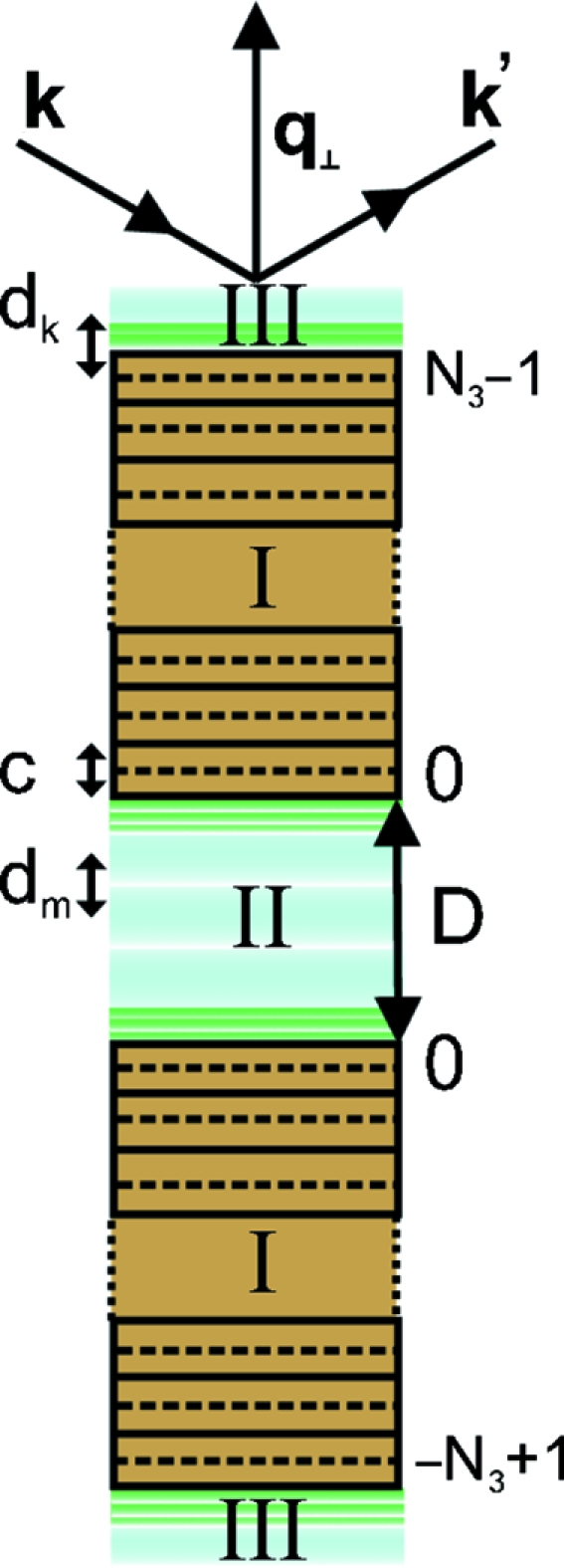
Variables used for the calculation of the structure factor amplitude. Left-hand side: the liquid’s layer positions *d*_*m*_ away from the centre of the gap, *d*_*k*_ away from the outmost mica unit cell centre and *c* the mica unit-cell height are indicated with arrows. Right-hand side: the number of mica unit cells in each mica membrane is *N*_3_. The gap width *D* is defined as the distance between the surface potassium ions of the opposing mica crystals.

Fig. 3 ▶ illustrates the variables used for the calculations below. The total structure factor *F*(*q*_⊥_) for the confinement device is written as a sum of contributions from the regions I, II and III indicated in Fig. 3 ▶, 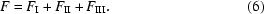 Here, *F*_I_ is the structure factor for the pair of columns of mica unit cells, *F*_II_ that of the confined liquid and *F*_III_ that of liquid condensed on the outer mica surfaces. The squared modulus (‘structure factor intensity’) is given by 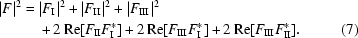
Below we provide theoretical expressions for these structure factor terms as well as for the interference terms 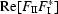 and 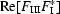. We will argue that 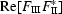 ≃ 0.

### 3.1. Structure factor for mirrored mica crystals at distance *D*

In the following derivation, surface roughness effects are neglected since the cleaved mica membranes are free of atomic steps. We start with the structure factor of a single mica unit cell,
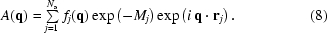
Here, *N*_a_ is the number of atoms in the unit cell, *f*_*j*_ the atomic form factor of the *j*th atom, **r**_*j*_ its position and exp(−*M*_*j*_) the Debye–Waller factor. Unit-cell dimensions, atom positions and r.m.s. thermal displacements for muscovite mica were taken from Güven (1971 ▶) and Schlegel *et al.* (2006 ▶). From now on we assume the origin of the unit cell to be in the plane going through its centre and we use the approximation **q**·**c** = *qc*cos(5.76°) = 0.995*q*_⊥_ *c* ≃ *q*_⊥_ *c*. The structure factor of two mirrored mica crystals at distance *D*, with each crystal represented as a stack of *N*_3_ unit cells (Fig. 3 ▶), can then be expressed by the following sum of phase factors,
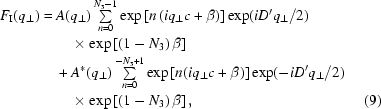
where *D*′ = *D* + *c* is the distance between the centre planes of the unit cells bordering the gap and exp(−β) is the amplitude attenuation factor for a single unit cell within the stack. The latter can be determined from β = *c*/(Λsinθ), where Λ is the 1/*e* attenuation length for the beam intensity (Λ = 677 µm at a photon energy of 16.5 keV). Summing the geometric series we obtain 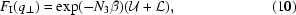
with the structure factors 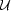 and 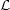 for the upper and lower stacks given by
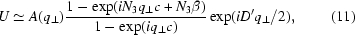

               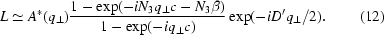
Because the attenuation by a single mica unit cell is very weak for non-grazing angles of incidence, we have simplified the nominators of the sums 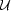 and 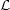 in (11) and (12) by approximating exp(±β) ≃ 1. By contrast, with the number of unit cells in a single stack being typically *N*_3_ = 4000, the attenuation factor after traversal of the stack is in the range 0.7 < exp(−*N*_3_β) < 0.9 for incidence angles 1 < θ < 4°.

The structure factor intensity equals 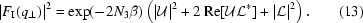
Substitution of the expressions for 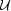 and 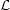 yields many terms, of which the ones containing exp(±*iq*_⊥_ *N*3*c*) or exp(±2*iq*_⊥_ *N*3*c*) are rapidly oscillating. Because the period 2π/(*N*_3_ *c*) ≃ 10^−4^ Å^−1^ of these oscillations is much smaller than the experimental momentum resolution Δ*q*_⊥_ ≃ *q*_⊥_(Δθ) ≃ 4 × 10^−3^ Å^−1^, they are not resolved and average out to zero. Dropping the fast oscillating terms and assuming *A*(*q*_⊥_) = A*(*q*_⊥_) (the mid-plane of the unit cell is the symmetry plane), we derive the simple expression 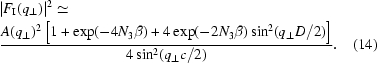
The first two terms are the well known expressions for crystal truncation rods (CTRs) (Robinson, 1986 ▶), in our case resulting from the crystal truncations at the upper and lower outer mica surfaces. The CTRs effectively scatter incoherently because the interference fringes associated with twice the mica thickness are not resolved. The third term describes the interference between the CTRs from the two inner surfaces at distance *D*. Note that the limiting case of zero gap (*D* = 0) just yields the two independent CTRs from the outer surfaces, as expected.

### 3.2. Structure factor for layered liquid in the gap

The layered electron density distribution within the gap shall be represented as a series of Gaussian peaks symmetric with respect to the gap centre. Denoting the width of layer *m* at position *d*_*m*_ (Fig. 3 ▶) by σ_*m*_ we write the electron density distribution as a function of distance *z* from the gap centre (*z* = 0) as 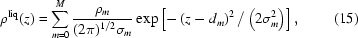
where *M* is the number of Gaussian peaks (layers) in the gap and ρ_*m*_ is the areal electron density of each layer. The structure factor of the liquid in the gap is obtained by Fourier-transforming the electron density distribution and accounting for the attenuation of the amplitude through the upper mica membrane,
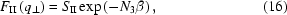
with 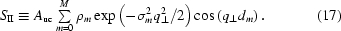
The corresponding structure factor intensity becomes 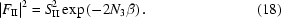

            

### 3.3. Structure factor for liquid condensed on the outer mica surfaces

The liquid adsorbed on the outer mica surfaces is also modelled as a series of Gaussian peaks and the liquid layers are again assumed to be positioned symmetrically with respect to the gap centre. The corresponding structure factor is of the form
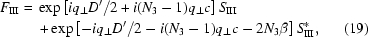
where the factor exp(−2*N*_3_β) accounts for the attenuation of the amplitude by the two mica membranes. *S*_III_ is the Fourier transform of the density,
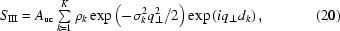
with *d*_*k*_ being the adsorbed layer positions relative to the centre of the outermost mica unit cell (Fig. 3 ▶). After removal of the fast oscillating terms we obtain for the structure factor intensity
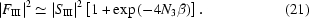

            

### 3.4. Interference terms

The interference between the amplitudes scattered from the liquid in the gap and from the mica crystals is described by the term
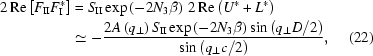
where again the fast oscillation terms have been put to zero. Similarly we find for the term describing the interference between scattering from the liquid adsorbed on the outer mica surfaces and from the mica crystals
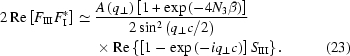
The product 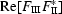 only contains exp(±*iq*_⊥_ *N*_3_ *c*) terms, so that it averages out to zero for the momentum resolution in our experiment. Hence, the confined liquid and the liquid adsorbed on the outer surfaces scatter incoherently.

### 3.5. Total structure factor intensity

By rearranging the terms derived in the previous subsections we can write the expression for the total structure factor intensity as a sum of contributions from the inner and outer crystal terminations,
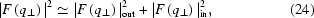
with
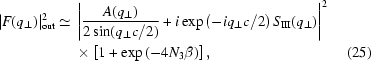

               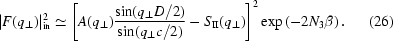
The first term, 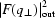, contains the amplitudes of the CTR and the liquid adsorbed on the outer surfaces of the upper and lower crystals, whereas the second term, 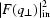, contains the amplitudes of a modulated CTR and the confined liquid (we recall that *A* and *S*_II_ are real, whereas *S*_III_ is complex).

It is interesting to consider various limiting cases of (26). For example, if we put *D* = *c* and we fill this gap with one unit cell of mica (*S*_II_ = *A*), the two opposing mica crystals are fused to one crystal and the second term 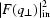 vanishes. Generally, the closer the similarity between the density profiles for the layered liquid and the mica crystal planes, the smaller is 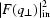. However, the liquid’s atoms generally have on average a much lower *Z*-number than those of mica, which causes this term to be significant. High sensitivity for the liquid’s density profile is expected near the mica Bragg reflections at *q*_⊥_ = 2π/*c* if the interlayer distance of the liquid is close to the mica unit-cell height *c*. Below, we examine such a case.

## 4. Determination of the density profile

**Figure 4:**
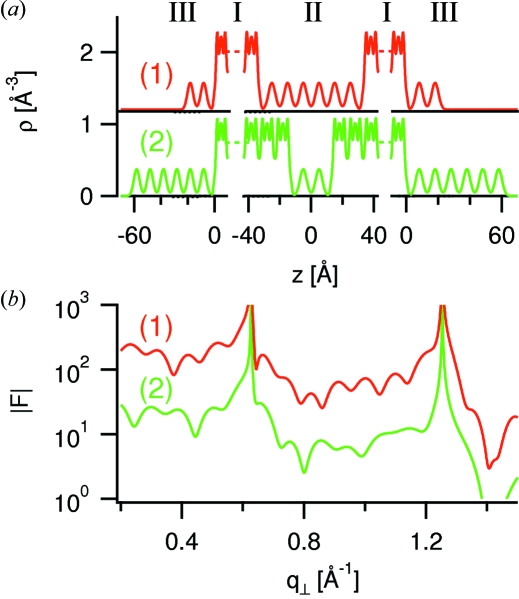
(*a*) Electron density profiles for models (1) and (2) in which the confined and adsorbed liquids are exchanged. (*b*) Structure factor amplitudes calculated for the model electron density profiles (1) and (2) of panel (*a*). The second curve has been shifted downwards for better display.

Confined liquid can be distinguished from liquid adsorbed on the outer surfaces because their structure factors *S*_II_ and *S*_III_ contribute to the total structure factor intensity in a different way. Namely, the interference term between *A* and *S*_II_ in 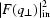 [equation (26)] is modulated by the factor 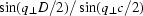, whereas the interference term between *A* and *S*_III_ in 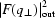 [equation (25)] is modulated by 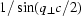. For illustration we consider the two model profiles shown in Fig. 4(*a*) ▶, in which the liquids are represented as sequences of molecular layers of equal height and width at equal distances of 1 nm. One profile has six molecular layers of confined liquid within a gap of 6.6 nm and two layers of adsorbed liquid; the other profile has these liquids exchanged (gap width of 2.6 nm). The corresponding structure factor amplitudes and intensities are shown in Figs. 4(*b*) ▶ and 5 ▶, with the latter showing the separate contributions from 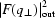 and 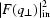 from the outer and inner regions, respectively. The interference fringes related to 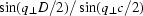 have a much shorter period for the first model owing to the larger gap than for the second model. This results in different *q*_⊥_-dependencies of the total structure factor. Our *a priori* knowledge of the gap width *D* through white-light interferometry provides a useful constraint to the number of plausible models for the confined and adsorbed liquids.

**Figure 6:**
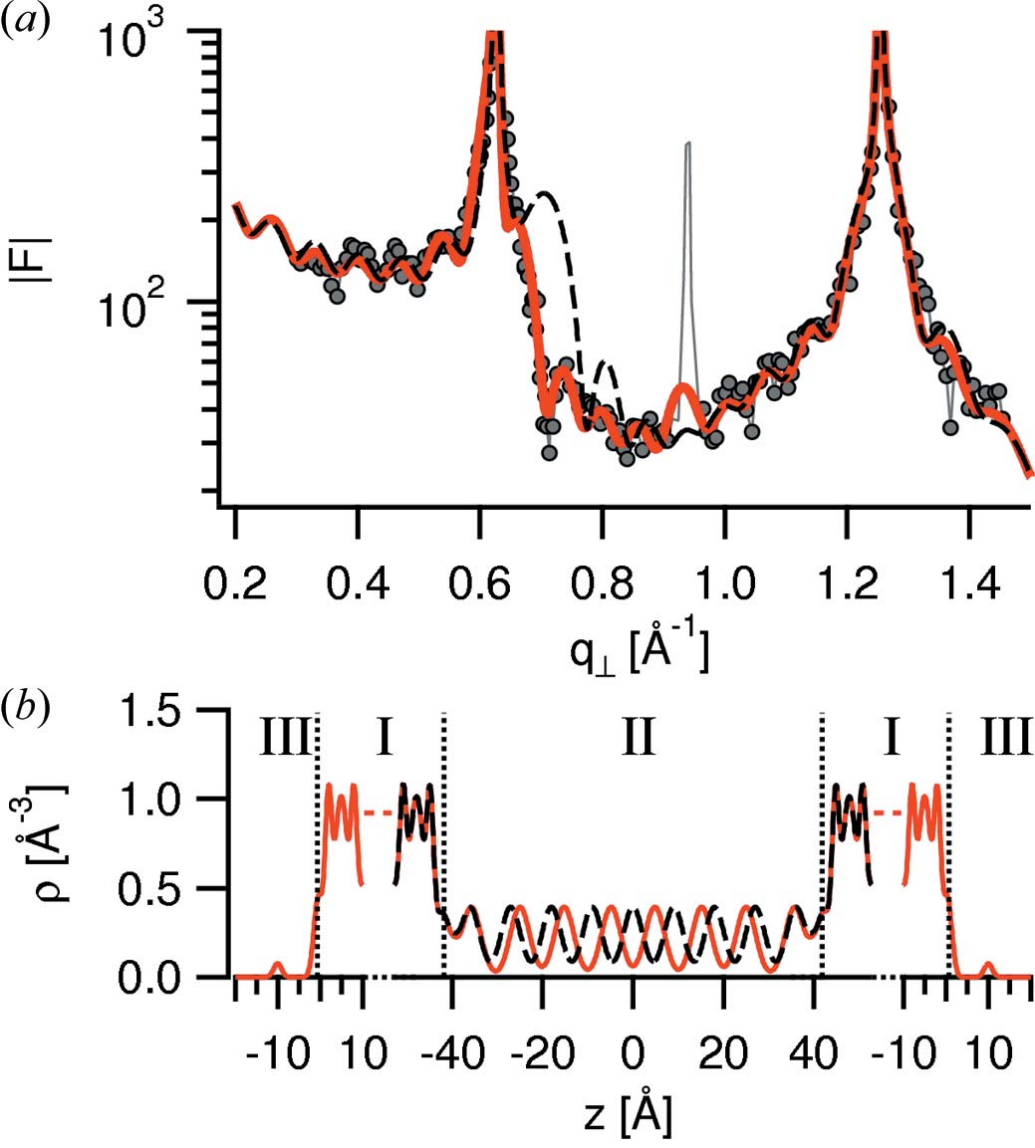
Measured and calculated structure factor amplitudes with corresponding electron density profile for TTMSS confined by mica membranes at a distance of 8.6 nm. (*a*) Measured amplitudes are indicated by the grey dots, amplitudes for the best-fit model by the red solid curve and amplitudes for a deviating model by the black dashed curve. (*b*) Corresponding best-fit and deviating electron density profiles are indicated by solid red and black dashed curves, respectively.

We now apply the reflectivity theory to the XRR data obtained for confined TTMSS. Specularly reflected intensities *I*_int_(*q*_⊥_) were integrated for momentum transfers *q*_⊥_ up to 1.4 Å^−1^. Using equation (3), with *f* initially set to 1, values for the corresponding structure factor amplitudes |*F*_exp_(*q*_⊥_)| were derived. Sets of calculated values |*F*_calc_(*q*_⊥_)| were generated for a variety of structure models for the confined and adsorbed liquids as discussed in §3. The measured and calculated sets of values were compared using the logarithmic residual (Hirano *et al.*, 1998 ▶) 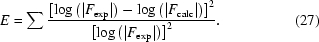
The model structural parameters, including the number of layers and *f*, were varied so as to minimize the residual *E* and thus to find the structure providing the best fit. In order to reduce the number of fitting parameters, the confinement arrangement was taken to be symmetric, the TTMSS layers in the gap were assumed to have equal electron density and width. The following additional constraints were applied: the liquid was not allowed to penetrate the mica, areal densities of the liquid layers were not to exceed the electron density for triangular closest packing of TTMSS molecules (calculated for a molecule diameter of 9.0 Å), and the width of the layers was kept to a lower limit of σ = 2 Å. In total, 23 fitting parameters were used: 12 symmetrical confined Gaussian peaks were fitted, each having a position (six parameters), a width and a height. The widths and heights of the inner eight density peaks were assumed to be equal, which results in two parameters plus four parameters from the boundary layers. The liquid on the outer mica surfaces were fitted with three layers, each having a position, a width and a height (nine parameters). Furthermore, the gap width and the correction factor *f* were two additional fitting parameters. We note that a number of fitting parameters are correlated, for example the width and the height of the Gaussian peaks. Fig. 6 ▶ shows the best-fit structure factor amplitudes in comparison with the measured values and the corresponding best-fit electron density profiles. All density profiles have been broadened with the experimental resolution (π/*q*_⊥,max_ = 2.2 Å) (Fenter, 2002 ▶). The best fit has been achieved for *E* = 0.30 and *f* ≃ 0.6.

The measured structure factor amplitude in Fig. 6(*a*) ▶ shows two Bragg peaks from the mica crystal planes and is modulated owing to interference effects between liquid and crystal as discussed before. From the sharpness of the mica Bragg peaks (FWHM < 0.01 Å^−1^) the bending of the confined film along the beam is estimated to be smaller than 1 mrad. Bending effects are therefore thought to be negligible. The peak at ∼0.9 Å^−1^ results from stacking faults in the mica and has therefore not been fitted. The sensitivity of the amplitude modulations to the film thickness and the structural parameters of the liquid such as the electron density amplitude and the interlayer distance has been discussed by Perret *et al.* (2009 ▶). Here we illustrate the sensitivity of the modulations to the liquid’s interlayer distance. The pronounced broad maximum under the first mica Bragg peak at *q*_⊥_ = 0.63 Å^−1^ indicates that the confined liquid is layered with a period about equal to the height of the mica unit cell (∼10 Å). By contrast, a model electron density profile [dashed black curve, Fig. 6(*b*) ▶] with liquid layers at distances equal to the molecular diameter (9 Å) leads to a broad peak in the modulus structure factor at a larger momentum transfer than is experimentally observed.

**Figure 7:**
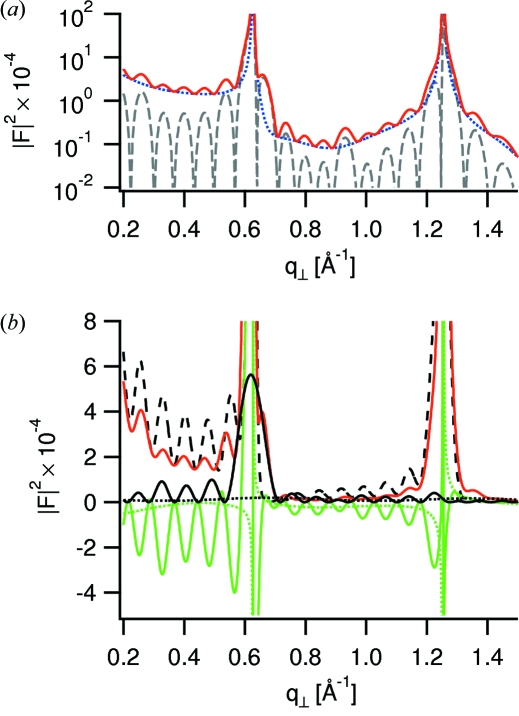
Decomposition of best-fit structure factor intensity (red solid curve) into contributions from different regions of the confined system. (*a*) Decomposition into contributions from the inner and outer regions according to equation (24). Blue dotted curves display 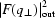, grey dashed curves 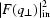. (*b*) Decomposition into the individual terms of equation (7). 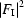, dashed black curve; 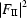, solid black curve; 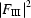, dotted black curve; 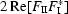, green solid curve; 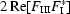, green dotted curve.

A decomposition of the best-fit structure factor intensity in contributions from the inner and outer crystal regions is shown in Fig. 7(*a*) ▶. The contribution from the outer regions, 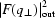, follows the typical shape of a CTR, modulated by the presence of an adsorption layer. The contribution from the inner region, 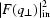, displays fringes with a period equal to the inverse gap width, 2π/*D*. A further decomposition into the individual terms of equation (7) is shown in Fig. 7(*b*) ▶. The structure factor intensity for the opposing mica crystals, |*F*_I_|^2^, is modulated with a period reflecting the gap width and contains the pronounced mica Bragg peaks. The structure factor intensity for the confined liquid, |*F*_II_|^2^, shows a peak at the inverse interlayer distance below the first mica Bragg peak and is as well modulated with a period equal to the inverse gap width. The structure factor intensity for the outer liquid, |*F*_III_|^2^, exhibits slow modulations and is negligible in this case, because only a small amount has been adsorbed. For the same reason, the interference term 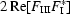 is small away from the Bragg peaks. The interference term 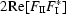 oscillates in anti-phase with |*F*_I_|^2^ and |*F*_II_|^2^.

## 5. Conclusion and outlook

An X-ray reflectivity theory has been presented for retrieval of the density profile of liquid confined between two opposing crystals. Use has been made of the interference between the scattered amplitudes from the crystal planes (of known structure) and the layered density profile of the liquid (unknown). The theory has been applied in order to analyse reflectivity curves from TTMSS confined by cleaved single-crystal mica membranes, and the liquid’s density profile has been determined. The theory can be readily extended to non-specular reflectivity from a pair of equally oriented crystal lattices confining a liquid. This would in principle enable a full determination of the molecule’s positions within a planar crystal void of adjustable thickness. Such a confining geometry, if it can be experimentally realised, would be ideally suited for structural studies of confined water.

## Figures and Tables

**Figure 5:**
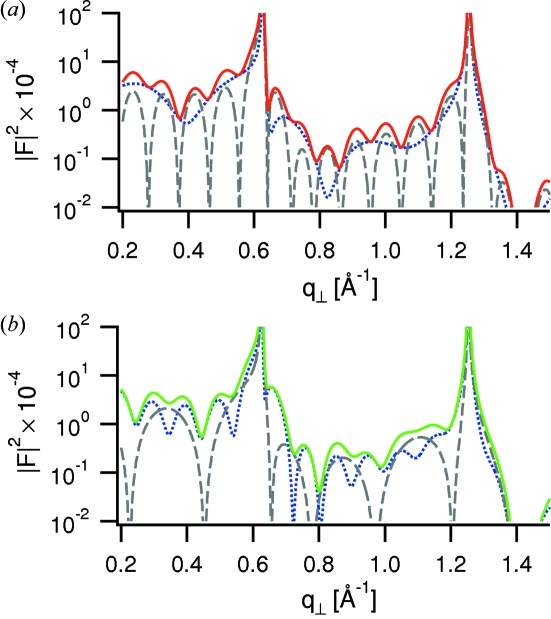
Structure factor intensities calculated for the two models displayed in Fig. 4 ▶. Blue dotted curves display the contribution 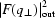 from the outer regions, grey dashed curves the contribution 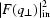 from the inner regions. (*a*) Model (1), (*b*) model (2).
